# New technology to improve the thermal stability of botulinum toxin type D by biomimetic mineralization

**DOI:** 10.1038/s41598-021-83733-9

**Published:** 2021-02-18

**Authors:** Shengqing Li, Xiyun Zhang, Guoyuan Hu, Shuping Li, Zhining Li, Yuxia Fan, Yanming Zhang

**Affiliations:** 1 Northwest Agriculture and Forest University, Yanglin, 712100 China; 2 Qinghai Academy of Animal Science and Veterinary Medicine, Qinghai University, Xining, 810016 China

**Keywords:** Biological techniques, Chemical biology, Developmental biology, Microbiology, Medical research, Molecular medicine

## Abstract

The advanced biomimetic mineralization technology was applied to protect the *Botulinum* neurotoxin type D, and the processing of the mineralization granule of botulinum toxin type D was successfully screened. The loss of activity of the toxin protein at different temperatures and the destructive strength of the gastrointestinal tract against the toxin were determined biologically. The lethal toxicity of the mineralized toxin to wild rodents was determined by median lethal dose. Protective tests at different temperatures showed that the preservation period of botulinum toxin type D mineralized sample 2 was significantly higher than that of the control group at three different temperatures, and its toxicity loss was significantly reduced. The damage intensity of the mineralized toxin to the gastrointestinal contents of plateau zokor and plateau pika was significantly reduced. The minimum lethal doses of the mineralized toxin particles to plateau zokor, plateau pika, and mice were 5200, 8,600,000, and 25,000 MLD/kg. These results showed that biomimetic mineralization could greatly improve the thermal stability of botulinum toxin type D and reduce the damaging effect of the gastrointestinal contents of target animals to botulinum toxin type D. The mineralized toxin could be used to control the population density of urban rodents. This research provides new insights into the protection of toxin protein substances.

## Introduction

type botulinum rodenticide is a biological rodenticide developed by *Clostridium botulinum* toxin type D. This strain was first isolated from animal carcasses in 1989 in China^1,2^. Botulinum toxin type D has a high targeted killing efficiency on plateau pika and plateau zoker in the plateau pastoral area. This rodenticide has achieved efficient outcomes on the prevention and control of three main pests in the Qinghai–Tibet Plateau (plateau pika, plateau zokor, and Qinghai voles)^3,4^. However, this protein toxin is easily degraded and inactivated by ultraviolet light and sunlight. In addition, the toxin has a strong destructive effect on the intestinal contents of rodents, thus exacerbating the difficulty in storing and transporting this toxin and limiting its application range.

Traditional protein protection methods generally involve cryopreservation or freeze-drying technology. Although they could prolong the shelf life of the protein toxin to a certain extent, they are time consuming and labor intensive, and the protein toxin preservation effect is unsatisfactory. Researchers attempted to imitate the structure, morphological characteristics, and function of biological materials to gradually establish the concept of material bionics and solve problems associated with protein protection. Chang first proposed the concept of “artificial cells” in 1964 and applied it to the medical field^5^. Microcapsules with a similar size of biological cells could be prepared by mimicking the structure of cells and being embedded with islets. These microcapsules could solve the host’s immune graft rejection problem. In 2013, a research team in Zhejiang University worked closely to improve the thermal stability of vaccines^6^ on the basis of the protective function of natural eggshells. The bio-mineralization construction of a shell that mimics an eggshell for vaccines initially improves the thermal stability of vaccines through the biomimetic modification of Japanese encephalitis virus vaccine. This mineralized shell gives vaccines an efficient thermal stability for them to be stored at 26 °C for more than 9 days at 37 °C for approximately 1 week^7,8^. Botulinum toxin type D is composed of a light chain (L chain, amino terminal, 50 kDa) and a heavy chain Hc (H chain, carboxyl end, 100 kDa). This protein is linked by a disulfide bond^9^. The Hc fragment is the main antigenic fragment of botulinum toxin^10^. In addition, botulinum toxin has properties similar to those of vaccine antigen proteins, indicating that the principle of biomimetic mineralization could be applied to obtain good thermal stability. In the present study, biomimetic mineralization was applied to screen three kinds of bio-based materials and the biomimetic mineralization technique of botulinum toxin type D protein was established. The protective effect of the mineralized toxin at different temperatures, the overflow efficiency of active ingredients, and the damage intensity of the mineralized toxins to the intestinal tracts of different animal models were characterized. The median lethal dose (LD_50_) of the mineralized toxins to different rodents was also determined.

## Materials and methods

### Materials

#### Experimental animals

Wild plateau pika (*Ochotona curzoniae*) with an average body weight of 120 g was captured in Haibei County, Qinghai Province. Wild plateau zokor (*Eospalax fontanierii*) with an average weight of 150 g was captured in Datong County, Xining City, Qinghai Province. Sprague–Dawley rats were purchased from the Qinghai Provincial Institute of Endemic Diseases.

#### Animals ethical statement

All animal procedures were performed according to guidelines laid down by the China Council on Animal Care, and the protocol was approved by the Experimental Animal Manage Committee of Qinghai University.

#### Medicine

The following substances were used: botulinum toxin type D (provided by the Laboratory of Qinghai Animal Husbandry and Veterinary Sciences, No. 20171008), methylcellulose (MC) sodium salt, sodium carboxymethylcellulose, gelatin, sucrose, sodium alginate (SA), protamine sulfate, sodium silicate, talc, Tween-20, and other reagents and chemicals (Tianjin Kaitong Chemical Reagent Co., Ltd).

### Methods

#### Ethical statement

Ethics Committee approval was obtained from the Institutional Ethics Committee of Qinghai University prior to the commencement of the study.

#### The statement confirms

All experiments were performed in accordance with relevant guidelines and regulations. And the authors complied with the ARRIVE guidelines.

##### Preparation of botulinum type D biomineralized toxin particles

MC, sodium carboxymethylcellulose, carboxymethyl chitosan, and 10% sucrose plus 30% gelatin solution were selected as bioprotective agents for botulinum toxin type D. The test was performed to determine the concentration of each substance and the mixing ratio.

Biomineralization synthetized the protected shell membrane of botulinum toxin type D. Comparative experiments were performed to determine the concentration of sodium alginate solution when the capsule was wrapped, the concentration of calcium ions in the mixed solution, the coating time of the capsule, the calcification time, the oscillation frequency, and other technical parameters. These parameters were determined to observe the shape and size of the mineralized particles under different conditions, toxin stability, overflow rate, capsule thickness, and other characteristics and identify the optimal capsule coating process.

Mineralization generated the hybrid shell wall of botulinum toxin type D. A contrast test was conducted to determine the optimal concentration of protamine solution and sodium silicate solution in alginic acid–protamine (AP) microcapsule hybridization, and the hybridization time was observed. The hybridization traits under different conditions were detected.

##### Storage stability of three mineralized toxin samples at different temperatures

The biomineralized botulinum toxin type D samples 1, 2, and 3 and the pure toxin control samples prepared for the test were exposed to 22 °C, 37 °C, and 55 °C, respectively. Under each temperature condition, each biomineralized botulinum toxin type D sample was exposed to a certain temperature on days 7, 14, and 30. Then, the samples were exposed monthly. The toxin in the mineralized granules was determined through intravenous injection to mice.

##### Oral toxicity of different mineralized toxin particles on mice

Biomineralization was applied to produce botulinum toxin granules with virulence of 100,000; 50,000; 20,000; and 10,000 MLD/particle. Kunming mice (18–22 g) were divided into four groups, namely, A, B, C, and D, and each group had five animals fasted for 12 h before they were orally administered with one of the four mineralized toxin particles. The death of the mice was observed, and the optimal toxin ratio was determined.

##### Lethality test of mineralized toxin on plateau pika and plateau zokor

Biomimetic mineralization was utilized to prepare mineralized particles with toxic contents of 2000, 1000, 500, and 250 MLD/particle. The plateau pika and plateau zokor captured in the wild field were divided into four groups, namely, I, II, III, and IV, and each group had five individuals fasted for 12 h before they were orally treated with the mineralized toxin. The four mineralized toxin granules were placed, and the deaths of plateau pika and plateau zokor were observed.

##### Comparative analysis of the damage intensity of biomineralized botulinum toxin type D to the intestinal contents of different rodents

After the plateau Zokor and plateau pika were sacrificed, 1.0 g of intestinal contents was immediately collected and placed in two centrifuge tubes and mixed with 4 mL of normal saline. One tube was added with mineralized toxin with a toxicity of 500,000 MLD/mL. The other tube was added with pure toxin and fully mixed. After 24 h, the amount of residual toxin was determined in 18–22 g Kunming mice.

## Results

### Technical flow of biomimetic mineralization of botulinum toxin type D

MC, hydroxypropyl MC sodium, and gelatin sucrose were mixed as a protective agent. In the test, the optimal concentration of the protective agent was 2.0%, and the optimal molding effect was observed when the ratio of toxin:protectant:CaCl_2_ was 1:3:1. If the concentration of the protective agent was too low, the droplets were trapped on the surface of the solution, forming a hemispheric shape and causing the toxin protein to overflow. If the concentration of the protective agent was too high, a pear-shaped microcapsule was easily formed, the tip was easily broken, and the toxin overflowed easily.

**Figure 1:**
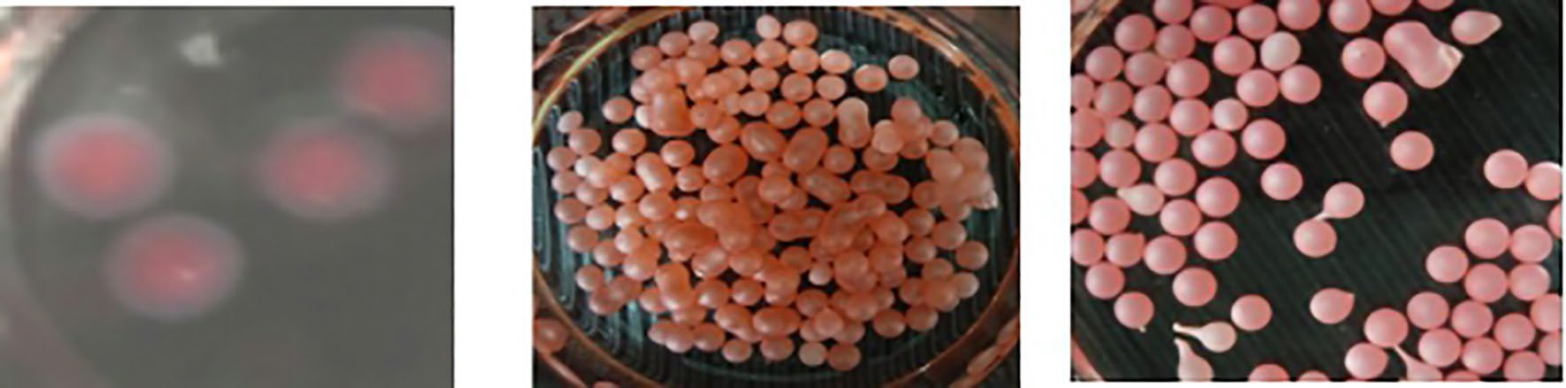
(**1-1**) Appearance of calcified microcapsules of botulinum toxin type D; (**1-2**) Microcapsule after alginate/protamine wrap; (**1-3**) Alginic acid/protamine/silica.

In the biomineralization to synthetize the protected shell membrane of botulinum toxin type D, 5 mL of the botulinum neurotoxin type D (BoNT)-containing solution mixed with different protective agents was separately aspirated with a disposable syringe and dropped into 40 mL SA solution (1.0% w/v) through a needle with an inner diameter of 0.45 mm. Immediately after the solution came in contact with the SA solution, the liquid core solution formed a calcium alginate gel shell membrane by cross-linking the Ca^2+^ and SA reaction. The SA solution was continuously stirred to prevent the microcapsules from sticking. After gelation occurred for 30 min, 160 mL of deionized water was added for dilution, and the solution was gently stirred for 5 s. The microcapsules were filtered with a gauze and washed two times with 10 mL of Tris-HCI buffer solution to remove the SA remaining on the surface of the capsule. The microcapsules were immersed in 10 mL of CaCl_2_ solution, magnetically stirred for 10 min, filtered with a gauze, and washed two times with buffer solution to remove Ca^2+^ on the outer wall of the microcapsules and improve the mechanical strength of the shell membrane. The mineralized toxin particles of botulinum type D coated with calcium alginate shell film were obtained, as shown in Fig. 1(1-1), where the red part is the toxin-protecting agent mixture, and the white part is the protecting shell membrane.

The mineralized microcapsules embedded with BoNT were immersed in 35 mL of a 2 mg/mL protamine solution, shaken at 80 r/min on a shaker for 10 min, and filtered to obtain AP microcapsules, as shown in Fig. 1(1-2). The microcapsules were then immersed in 70 mL of a 30 mmol/L sodium silicate solution freshly prepared and adjusted to pH 7.0 with HCl, acted at a rate of 80 r/min for 15 min, and filtered and washed with a buffer solution to obtain alginic acid/protamine/silica (APSi) microcapsules [Fig. 1(1-3)].

### Storage period test of three mineralized toxin samples at different temperatures

**Figure 2:**
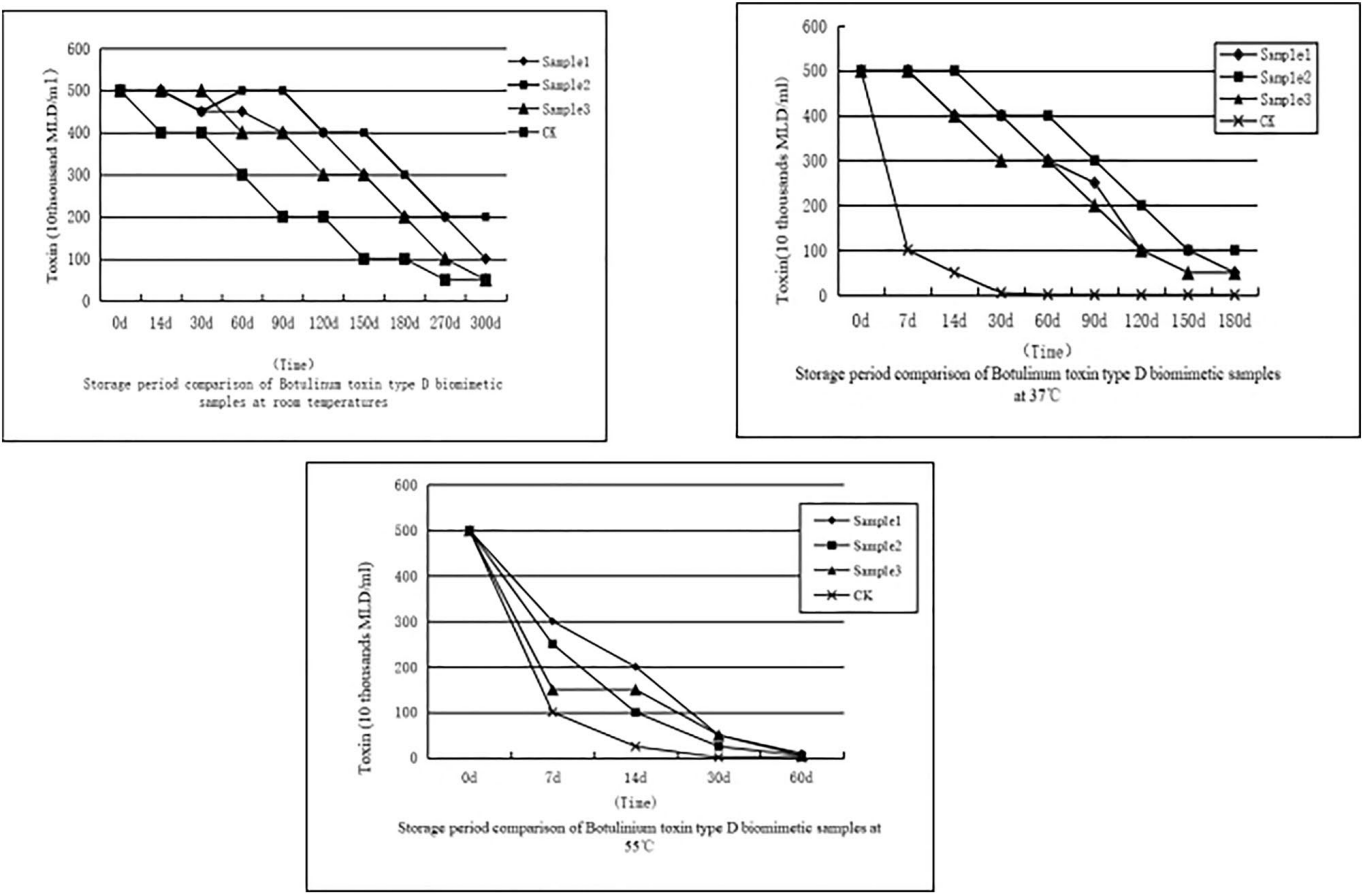
Comparison of storage stability among three mineralized toxin samples at different temperatures. (**2-1**) under 22 °C, (**2-2**) under 37 °C, and (**2-3**) under 55 °C.

At room temperature, the toxicity of the mineralized toxin remained unchanged for 3 months, whereas the pure toxin retained only 2/5 of the original toxicity [Fig. 2(2-1)]. At 37 °C, the toxicity of the mineralized toxin was maintained for 14 days, and the half-life was 3 months, but the virulence of the toxin not processed through biomimetic mineralization was only 1/5 of the original virulence after 7 days [Fig. 2(2-2)]. The mineral toxicity of the toxin exposed to 55 °C was maintained for 7 days. The residual toxin was 3/5 of the original virulence, whereas the control group was only 1/5 [Fig. 2(2-3)]. The experimental results showed that the storage life of the mineralized treatment sample 2 of botulinum toxin type D was significantly higher than that of the control group under three different temperature conditions, and its virulence loss significantly decreased.

### Oral toxicity test of different mineralized toxin granules in mice

The mice were fed with mineralized toxin particles placed at room temperature for 1 week. The oral test revealed that all of the mice in the group fed with 10,000 MLD/particle died (Table 1). This observation was consistent with the virulence evaluation standard of the product (protoxin), indicating that the toxin protein did not lose its toxicity during mineralization.

**Table 1:** Oral toxicity test of different toxic mineralized toxin particles on mice.

Group (10,000 MLD/each)	Number	Number of grains	Intake of toxin (10,000 MLD/kg)	Death
10.0	5	1	10.0	5/5 (24 h for all)
5.0	5	1	5.0	5/5 (24, 24, 24, 24, and 48 h)
2.0	5	1	2.5	5/5 (24, 24, 48, 48, 48, and 72 h)
1.0	5	1	1.0	3/5 (48, 48, and 72 h)

### Lethality test of the mineralized toxin on plateau pika and plateau zokor

In the oral test, all the plateau zokors in the group fed with 1000 MLD/particle (depending on the toxic dose of 5200 MLD/kg) died within 72 h (Table 2). All the plateau pika in the group fed with 1000 MLD/particle (depending on the toxic dose of 8600 MLD/kg) also died within 72 h (Table 3). These results indicated that the toxin virulence was stable after mineralization treatment.

**Table 2:** Oral lethality test of different toxic mineralized toxin particles on plateau zokor.

Group	MLD	Weight (g)	Average weight (g)	Intake of toxin (10,000 MLD/kg)	Death
I	2000	143.8	155.2	1.29	24 h
		176.5			24 h
		134.7			24 h
		168.3			24 h
		152.5			24 h
II	1000	186.8	191.4	0.52	24 h
		228.5			72 h
		162.1			48 h
		206.2			56 h
		173.6			60 h
III	500	202.6	193.9	0.26	Survived
		203.3			Survived
		133.9			Survived
		282.9			96 h
		146.9			Survived
IV	250	178.9	175.5	0.14	Survived
		186.3			Survived
		168.5			Survive
		189.9			Survived
		153.8			Survived

**Table 3:** Oral lethality test of different toxic mineralized toxin particles on plateau pika.

Group	MLD	Weight (g)	Average weight (g)	Intake of toxin (10,000 MLD/kg)	Death
I	2000	143.8	124.14	1.61	24 h
		106.5			24 h
		114.3			24 h
		128.3			24 h
		127.8			24 h
II	1000	113.6	115.78	0.86	50 h
		121.7			48 h
		108.5			48 h
		116.6			63 h
		118.5			48 h
III	500	108.9	115.24	0.43	Survived
		120.3			60 h
		114.6			72 h
		111.7			Survived
		120.7			76 h
IV	250	124.8	121.34	0.21	Survived
		136.7			Survived
		113.8			Survived
		106.8			Survived
		124.6			Survived

### Analysis of damage intensity in rodents’ intestinal contents with mineralized toxin treatment

The experiment showed that mineralization elicited an obvious protective effect on botulinum toxin type D. The virulence of three mineralized samples in the intestinal contents of plateau pika decreased from 400,000 MLD/mL to 250,000 MLD/mL. The toxicity of the original toxin decreased from 400,000 MLD/mL to 100,000 MLD/mL. Similarly, the virulence of mineralized samples 1 and 2 in the intestinal contents of plateau zokor decreased from 400,000 MLD/mL to 250,000 MLD/mL. The virulence of sample 3 decreased from 400,000 MLD/mL to 100,000 MLD/mL. The toxicity of the control group decreased from 400,000 MLD/mL to 50,000 MLD/mL (Table 4). These experimental results demonstrated that the damage of the intestinal contents to botulinum toxin was reduced after botulinum toxin type D was subjected to biomimetic mineralization**.**

**Table 4:** Comparative test of the destruction strength of mineralized toxins and pure toxins in the intestinal contents of two kinds of rodents.

Rodent	Plateau pike	Plateau zokor
	Toxin (10,000 MLD/ml)	
**Sample**		
Sample 1	25	25
Sample 2	25	25
Sample 3	25	10
CK	10	5

## Discussion

**Figure 3:**
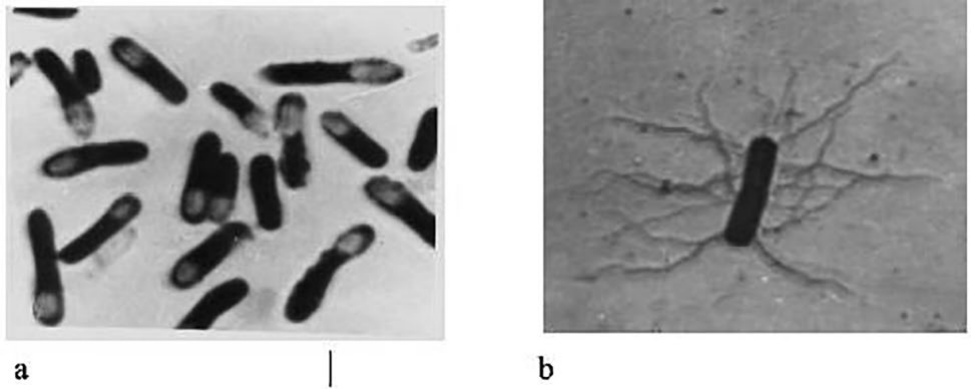
(**a**) Morphological characteristics, (**b**) electron micrograph of *Clostridium botulinum.*

**Figure 4:**
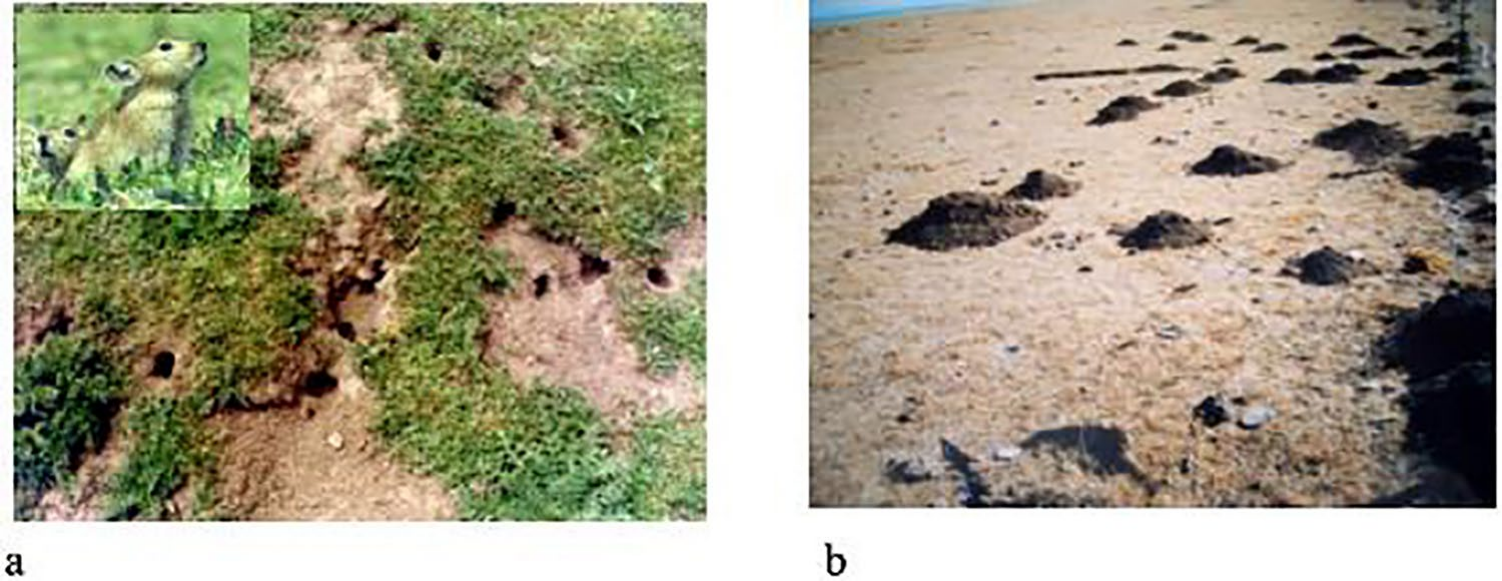
(**a**) Grassland damage caused by plateau pika, (**b**) Mound caused by plateau Zokor.

Since *Clostridium botulinum* was first isolated in 1897, seven toxin types, namely, A, B, C, D, E, F, and G, have been found^11^. Type D causes bovine and mutton poisoning in Africa, North America, and Australia. It has been isolated in China as well; one is from the East China Sea mud^2^, and the other is isolated in our laboratory from the carcass of a diseased sheep (D8901 strain) (Fig. 3). The application of botulinum toxin in cosmetic surgery is most prevail because it effectively treated crow’s feet^12^. This application has been used not only to reduce skin wrinkles in the mouth and neck^13^ but also for the benign hypertrophy of the masseter mandibular angle^14^. Botulinum toxin type D has also been applied to control grassland rodent in plateau pastoral areas (Fig. 4). It has achieved remarkable results in the prevention and control of grassland pests. However, it is a macromolecular protein that is sensitive to ambient temperature, ultraviolet light, and the environment of animals. Thus, this toxin could be easily degraded, which limits its scope to a large extent and increases the difficulty in storing it. In this study, the biochemical protection of botulinum toxin type D was achieved by biomimetic mineralization technology. The shelf-life test of the samples at different temperatures and the results of toxicity tests on different animals indicated that the use of biomimetic mineralization technology could change the nature of the toxin. It could also remarkably improve the tolerance of the toxin to temperature, thereby prolonging the residence time of the toxin in a wild and natural environment and greatly reducing the storage requirement of the toxin. These results provided a basis for using the toxin in a wide range.

The natural biomaterials found in nature have properties superior to those of artificial materials. Material bionics include structural bionics that mimic the structural characteristics of natural biomaterials and resemble material formation in organisms, bionics that mimic the composition of natural biomaterials, and functional bionics that mimic biological materials and system functions^15^. Among them, bionic silicification involving protein-based organic matter regulation is divided into four stages. First, the researchers found that silaffins extracted from diatoms could catalyze the formation of silicon oxide in vitro. Second, with the understanding of the chemical structure of silaffins, the researchers synthesized structural analogs of silaffins. Third, synthetic polymer polyamines, such as polyacrylamine and polyacrylamine hydrochloride, have been successfully used in bionic silicification^16–19^. Finally, natural proteins such as lysozyme, collagen, and gelatin have also been confirmed to catalyze bionic silicidation^20,21^. In the present study, to prevent the protective agent from negatively affecting the toxin protein, a bio-based material, such as methyl cellulose, was used as a liquid core protective agent for botulinum toxin protein and cross-linked with sodium alginate solution to form alginic. In the test, the concentration of the calcium alginate solution and the concentration ratio of the liquid core protective agent to the toxin protein seriously affected the formation degree of the mineralized particles, indicating that the concentration of Ca^2+^ seriously influenced the thickness of the membrane and the overflow efficiency of the toxin. The experiments verified that the best fit of concentration of the sodium alginate solution was 1.0%, and the ratio of toxin:protectant:Ca^2+^ was 1:3:1. The molding effect of this solution was the best, and the toxin loss rate was the lowest. The use of APSi-coated cystic toxin particles could enhance the mechanical tension of the capsule, improve the surface compactness, prevent toxin spillover, and be more conducive to the subsequent coating of granules.

Botulinum toxin protein has been used as a novel biological grassland pest control agent that does not cause residue formation, pollution, and secondary poisoning. It also does not adversely affect the characteristics of chemical rodenticides, the natural enemies of rats. However, as a macromolecular protein substance, it is highly sensitive to temperature and has limited scope and application of toxins. The biomimetically processed botulinum type D toxin granules showed a significantly improved tolerance for temperature. Meanwhile, the problems of requirements for the cold chain protection of toxins were solved. At room temperature, the toxicity of the mineralized treatment remained unchanged for 3 months, whereas the toxicity of pure toxin was only 2/5 of it. At 37 °C, the mineralization toxin toxicity remained unchanged for 14 days, and the toxin half-life was 3 months. However, the toxicity of botulinum toxin type D that has not been biomimetically processed was only 1/5 of the original virulence when stored in 37 °C for 7 days. At 55 °C for 7 days, the mineralized toxin retained 3/5 of the original virulence, whereas the control group had only 1/5 of the original virulence. The decline rate of the control group toxin virulence was significantly larger than that of the biomineralized toxin. The experimental results showed that the mineralized treatment method has significant protection for the botulinum toxin under three different temperature conditions. The protective effect of mineralization could significantly improve the tolerance of the toxin to temperature. This research greatly expanded the application scope of toxins from cold regions to hot regions.

Botulinum toxin is a biological rodenticide that effectively controls the population density of rodents and provides a post security for the treatment of black soil beach and the restoration of grassland vegetation^22–24^. However, the analysis of LD_50_ of botulinum toxin type D to different rodents has revealed that the sensitivity of the rodent to the toxin significantly differs^25,26^. For example, the minimum lethal dose of botulinum toxin in rats are 1,000,000 MLD/kg, much higher than that of plateau pika at 5000 MLD/kg. The present study examined the damage intensity brought by botulinum toxin type D to gastrointestinal contents. The results showed a positive correlation between the damage intensity of the toxin to different gastrointestinal contents and the sensitivity of the rats to the toxins, indicating that the destruction of gastrointestinal contents was one of the reasons for the decreased sensitivity of the rats to drugs. In this study, the damage intensity in the intestinal contents after biomineralized toxin treatment was significantly lower than that after pure toxin treatment, showing that biomimetic mineralization could significantly improve the bioavailability of botulinum toxin type D. This result provided a basis for using botulinum toxin to prevent rodents in cities and warehouses.
